# Choledochoduodenal fistula paradoxically prevents biliary obstruction: a case report

**DOI:** 10.3389/fsurg.2026.1752034

**Published:** 2026-04-20

**Authors:** Chengzhen Lyu, Ziqi Guo, Kun He, Xiyi An, Wenyi Deng, Huadan Xue, Gechong Ruan, Qingwei Jiang

**Affiliations:** 1 Department of Gastroenterology, State Key Laboratory of Complex Severe and Rare Diseases, Peking Union Medical College Hospital, Chinese Academy of Medical Sciences & Peking Union Medical College, Beijing, China; 2 Peking Union Medical College Hospital, Chinese Academy of Medical Sciences & Peking Union Medical College, Beijing, China; 3 Department of Radiology, Peking Union Medical College Hospital, Chinese Academy of Medical Sciences & Peking Union Medical College, Beijing, China

**Keywords:** biliary fistula, case report, choledochoduodenal fistula, cholelithiasis, protective fistula, recurrent choledocholithiasis, spontaneous stone expulsion

## Abstract

**Background:**

A 38-year-old woman with a 32-year history of recurrent biliary stones (cholecystectomy at age 6 and open choledochotomy with 6-month T-tube indwelling at age 16) presented with 2 h of postprandial right upper quadrant pain. Laboratory tests showed mild hyperbilirubinemia (total bilirubin 24.4 μmol/L, direct bilirubin 8.4 μmol/L) and elevated alanine transaminase of 287 U/L. Abdominal CT scan revealed common bile duct (CBD) stones without pneumobilia. Her symptoms resolved spontaneously before scheduled endoscopic retrograde cholangiopancreatography (ERCP). ERCP showed compensated dilatation of the CBD without residual stones, inadvertent contrast overflowing into the duodenum, a 5-mm choledochoduodenal fistula in the proximal descending duodenum, and a slender distal CBD segment confirmed by intraductal ultrasound. The fistula, further confirmed by enhanced CT, acted as a benign physiological drainage pathway. The slender distal CBD formed a specific pressure gradient, and spontaneous stone passage was achieved via this fistula, which was the core mechanism for the patient's long-term symptom-free survival.

**Conclusion:**

Choledochoduodenal fistula can, in rare circumstances, exert a protective rather than deleterious effect in patients with cholelithiasis. This case with a benign clinical course complements the clinical scenario beyond the conventional clinical paradigm that choledochoduodenal fistulas commonly require active intervention.

## 1 Introduction

Choledochoduodenal fistulas (CDFs) are uncommon, accounting for only 1.7–25% of all spontaneous internal biliary fistulas (1, 2). The vast majority (70–90%) stem from gallstone erosion, while 6–20% result from duodenal peptic ulcer disease; iatrogenic injury, malignancy, Crohn's disease, or tuberculosis are less common causes (3–8).

According to the established clinical paradigm, CDFs are almost uniformly pathological lesions, which usually require active intervention. The loss of the normal biliary-duodenal pressure gradient in most CDFs leads to enterobiliary reflux and pneumobilia, which presents clinically with recurrent ascending cholangitis, stone impaction and sump syndrome (6, 7, 9–11). Due to the persistent risk of reflux-related infectious and obstructive complications, current clinical guidelines and reviews overwhelmingly recommend active intervention (including endoscopic, surgical, or interventional procedures) even for minimally symptomatic CDFs.

Here, we report an exceptional case of benign CDF that protected a patient from recurrent cholelithiasis, and elaborate its unique pathophysiological mechanism and clinical implications.

## 2 Case presentation

### 2.1 Patient information and clinical history

A 38-year-old woman presented to our department with a 2-hour history of acute exacerbation of recurrent right upper quadrant abdominal pain. The pain was postprandial in onset and resolved spontaneously during transportation to the hospital. The patient had a 32-year history of recurrent biliary stone disease: she underwent open cholecystectomy for symptomatic gallstones at the age of 6. At 16 years old, she developed recurrent biliary colic; magnetic resonance cholangiopancreatography (MRCP) revealed multiple intrahepatic and extrahepatic bile duct stones, and upper gastrointestinal endoscopy showed no abnormalities. She then underwent open choledochotomy with T-tube drainage, followed by secondary stone extraction 3 months postoperatively, and T-tube removal 6 months after the choledochotomy. Over the subsequent 22 years, the patient remained completely asymptomatic with no documented stone recurrence until the current acute episode.

The patient had no history of smoking, alcohol consumption, or long-term medication use, and no family history of gallstone disease or hereditary disorders.

### 2.2 Diagnostic assessment

On physical examination, the patient's vital signs were stable. Mild tenderness was noted in the right upper quadrant, without rebound tenderness or a positive Murphy's sign.

Laboratory tests showed elevated liver function markers: alanine transaminase (ALT) 287 U/L, total bilirubin 24.4 μmol/L, and direct bilirubin 8.4 μmol/L.

**Figure 1:**
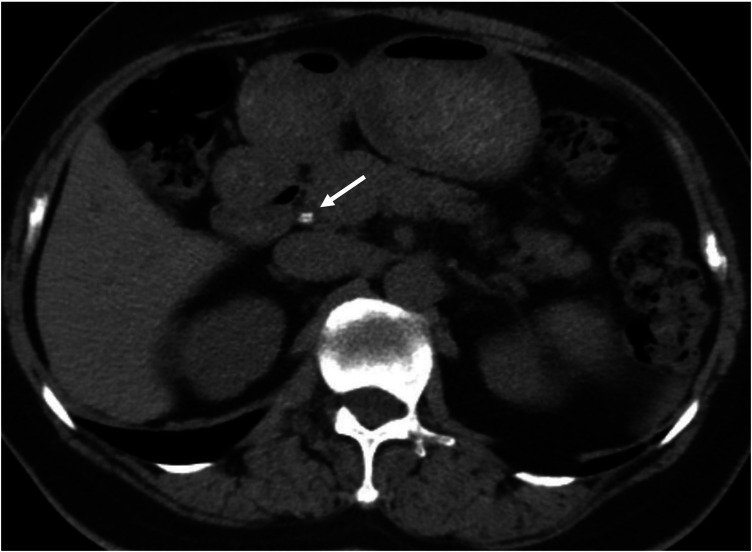
Axial view of the non-contrast abdominal CT scan on admission. The white arrow indicated the radiopaque stones in the common bile duct. No pneumobilia was observed in the intrahepatic or extrahepatic bile ducts.

Abdominal non-contrast computed tomography (CT) was performed, which demonstrated stones in the common bile duct (CBD), CBD dilatation, and an absence of pneumobilia (Figure 1). Endoscopic retrograde cholangiopancreatography (ERCP) was scheduled for etiological diagnosis and subsequent intervention.

**Figure 2:**
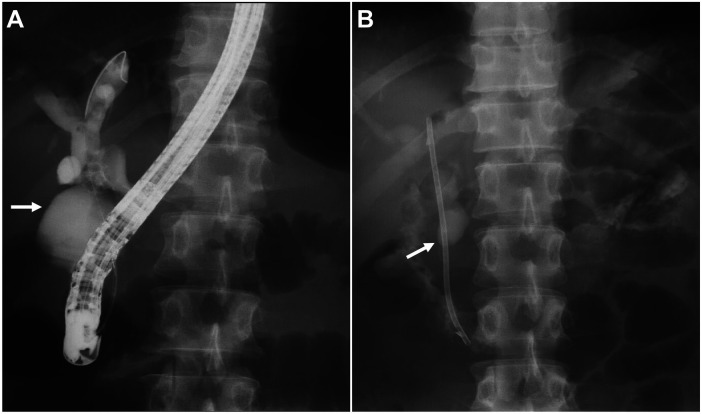
ERCP imaging findings. **(A)** ERCP image showed compensatory dilatation of the common bile duct, with no visible residual stones in the biliary tract. Inadvertent early opacification of the duodenal lumen (white arrow) was observed during contrast injection, which confirmed the presence of a choledochoduodenal fistula. **(B)** ERCP image after implantation of a 7 Fr × 12 cm plastic biliary stent for smooth drainage (white arrow). Contrast outflowing from the bile duct extended in the duodenum. ERCP: endoscopic retrograde cholangiopancreatography.

ERCP was performed after the patient's abdominal symptoms had partially resolved. Inadvertent early opacification of the duodenum was observed during contrast injection, indicating the presence of a biliary-enteric fistula (Figure 2A). The procedure revealed compensatory dilatation of the CBD, with no visible residual stones in the bile duct. Intraductal ultrasound (IDUS) further confirmed the absence of residual stones in the CBD and showed a slender distal CBD segment.

### 2.3 Therapeutic intervention

**Figure 3:**
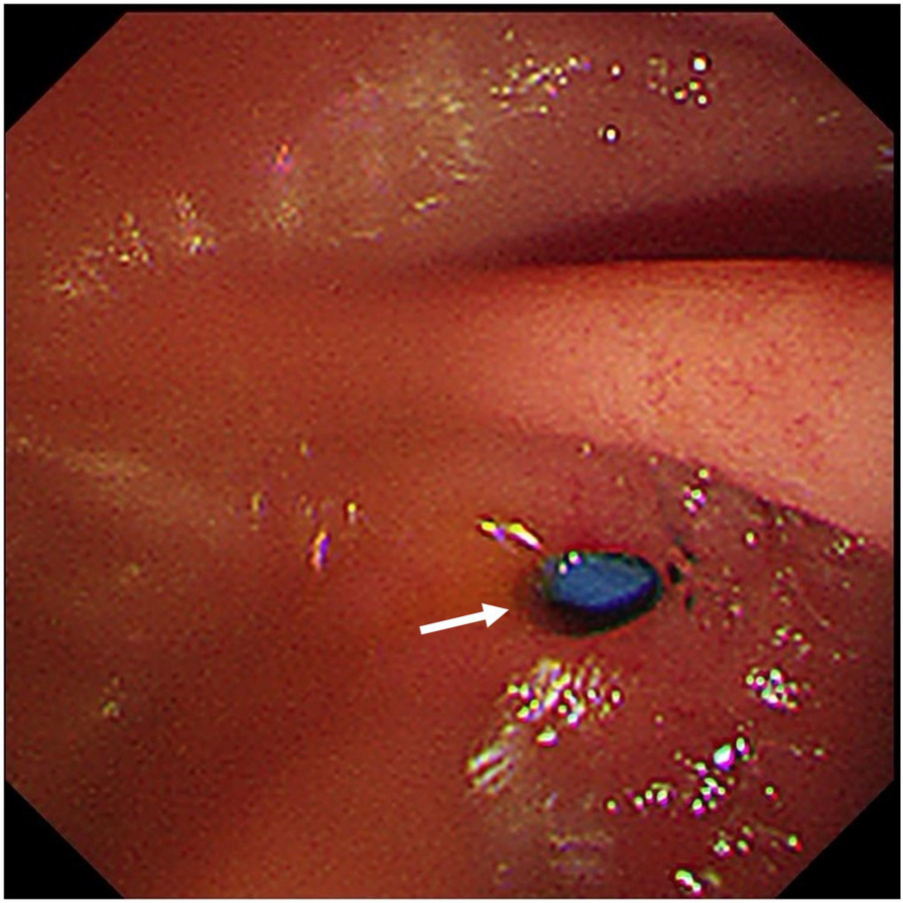
Duodenoscopic image obtained during scope withdrawal. A 5-mm fistula orifice (white arrow) was clearly visualized in the proximal descending duodenum.

To maintain long-term biliary drainage, a prophylactic 7 Fr × 12 cm plastic biliary stent was implanted during ERCP. Contrast injection after stent implantation again showed contrast overflow from the bile duct into the duodenum through the fistula (Figure 2B). During withdrawal of the duodenoscope, a 5-mm fistula orifice was clearly identified in the proximal descending duodenum, with the distal end of the implanted biliary stent protruding through the fistula into the duodenal lumen (Figure 3).

### 2.4 Follow-up and outcomes

**Figure 4:**
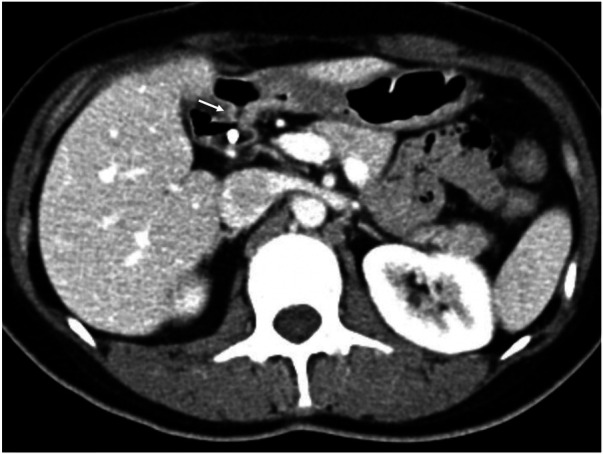
Axial view of post-ERCP enhanced abdominal CT detected the choledochoduodenal fistula in the proximal descending duodenum (white arrow), with implanted biliary stent. ERCP: endoscopic retrograde cholangiopancreatography.

Post-ERCP enhanced abdominal CT was performed, which confirmed the location of the choledochoduodenal fistula in the proximal descending duodenum and the *in situ* biliary stent (Figure 4). The patient had no procedure-related complications and was discharged uneventfully 2 days after ERCP.

One month after ERCP, the patient attended the outpatient clinic for routine follow-up, and denied any abdominal pain, fever, jaundice or other biliary tract-related symptoms since discharge. The biliary stent was successfully removed at this visit, with no adverse events during the procedure. Ursodeoxycholic acid was prescribed to promote bile excretion after stent removal.

During the subsequent two-year regular clinical follow-up, the patient remained consistently asymptomatic, with no recurrence or repeated episodes of biliary tract-related symptoms throughout the entire observation period.

## 3 Discussion

### 3.1 Conventional clinical paradigm of CDF

CDFs are traditionally classified into two major types based on location and etiology: type I (parapapillary, situated on the longitudinal fold of the papilla or immediately adjacent to it, usually secondary to distal choledocholithiasis) and type II (bulbar, on the posterior wall of the duodenal bulb, typically resulting from penetrating duodenal peptic ulcer) (3, 4, 6, 8, 12). Some authors have proposed a three-type system (supra-ampullary, periampullary, and infra-ampullary) (11), but the two-type classification remains the most widely accepted in clinical practice. Nearly all previously published CDF cases fall into the above classic classification framework, with the vast majority being spontaneous lesions caused by gallstone erosion or peptic ulcer penetration, and all reported cases are predominantly characterized by pathological damage to the biliary system.

More than 95% of CDFs are spontaneous, with gallstone disease (65–90%) and peptic ulcer disease (6–25%) being the dominant etiologies (7). Iatrogenic CDFs are exceedingly rare, accounting for less than 5% of all cases, and are almost always symptomatic due to biliary injury and subsequent reflux-related complications (5, 13, 14). The overwhelming majority of CDFs are considered pathological, as the fistula disrupts the physiological biliary-duodenal pressure barrier, leading to enterobiliary reflux, pneumobilia, recurrent ascending cholangitis, stone recurrence, and sump syndrome (7, 9, 10). Even in the few previously reported cases of asymptomatic incidental CDFs or transient relief of acute biliary obstruction via spontaneous stone passage through the fistula, pneumobilia and long-term risk of cholangitis are still universally present, and no case has documented a sustained long-term protective effect against biliary complications. For this reason, active intervention to close the fistula or manage its complications is the standard of care for nearly all CDF cases, while conservative management is rarely recommended.

### 3.2 Unique features and core protective mechanism

This case starkly deviates from the established clinical paradigm of CDF and nearly all previously published CDF cases, with three critical and unique features that underlie its long-term protective effect, among which the specific fistula location combined with the distal bile duct anatomical variation forms the core structural basis of this unique protective mechanism.

On the one hand, despite a fistula diameter of 5 mm, there was an absence of pneumobilia on pre-procedure imaging, with no evidence of pneumobilia during the prior 22-year follow-up. Pneumobilia is the hallmark of CDF in nearly all reported cases, present in 70–90% of patients, and indicates the loss of the normal positive biliary-to-duodenal pressure gradient (7, 15). The absence of pneumobilia in this case confirms that the physiological pressure gradient was fully preserved under baseline conditions, which is the fundamental prerequisite for preventing duodenobiliary reflux and recurrent cholangitis. In contrast, nearly all previously reported CDF cases—even those with incidental asymptomatic fistulas or transient stone passage events—are accompanied by persistent pneumobilia, which confirms the irreversible disruption of the physiological biliary-duodenal pressure barrier. These patients inevitably face a long-term high risk of recurrent cholangitis, thus requiring regular clinical monitoring or active invasive intervention even in the absence of acute symptoms.

On the other hand, the fistula is located in the proximal descending duodenum (supra-ampullary/peri-Vaterian) (14), a specific location that is rarely reported in previous CDF case studies, which avoids the direct damage to the duodenal papilla and sphincter of Oddi function seen in most periampullary CDF cases in published literature. Meanwhile, IDUS revealed a slender distal CBD segment that acts as a functional sphincter-like stricture (16, 17). This key anatomical feature is not described in previously published CDF cases, and it forms a significant and stable biliary-duodenal pressure gradient that is absent in other reported cases. Under physiological conditions, the baseline positive biliary pressure keeps the fistula functionally closed, preventing retrograde reflux of duodenal contents (11). When newly formed stones migrate into the CBD, the acute elevation of biliary pressure transiently opens the fistula. Meanwhile, the slender distal CBD increases the resistance of antegrade stone passage through the ampulla of Vater, so that stones are preferentially diverted through the lower-resistance fistula, achieving spontaneous expulsion into the duodenum (18). Unlike the transient stone passage effect occasionally reported in previous cases, which cannot form a long-term stable drainage mechanism and still requires active clinical intervention to prevent obstruction and reflux complications, this “pressure-dependent safety valve” mechanism in our case demonstrates the potential to achieve continuous and stable spontaneous stone expulsion while maintaining the long-term anti-reflux barrier of the biliary tract. This mechanism not only prevents biliary obstruction and stone incarceration, but also immediately normalizes biliary pressure to reclose the fistula, thus maintaining the long-term barrier function against reflux.

In addition, regarding the etiology of the fistula, it is possibly iatrogenic, secondary to the prior open choledochotomy and 6-month T-tube placement performed 22 years earlier. The patient's upper gastrointestinal endoscopy before the choledochotomy showed no abnormalities, and the long-term asymptomatic period began immediately after the surgery, which supports the above inference. This is distinct from the vast majority of reported CDF cases, which are spontaneous in origin (7).

Notably, while previous case reports have described transient relief of acute biliary obstruction via spontaneous stone passage through a CDF, these events are almost always isolated, transient phenomena (19–22). Previously reported cases still require repeated endoscopic or surgical interventions during follow-up, and patients often face repeated episodes of ascending cholangitis, which is fundamentally different from the 22-year symptom-free and intervention-free clinical course achieved by the stable protective mechanism in this case.

### 3.3 Clinical implications for management

This case expands the clinical spectrum of biliary-enteric fistulas, shifting the traditional perception that all CDFs are uniformly pathological lesions requiring active intervention. Our findings suggest that, in extraordinarily rare circumstances, a CDF can exert a biologically beneficial effect in patients with high-risk recurrent lithogenesis.

For patients with an established CDF, clinical decision-making should not be based solely on the presence of the fistula itself. Instead, it should be individualized based on the functional status of the fistula: the presence or absence of reflux, the risk of complications, and the underlying disease of the patient. For highly selected patients with recurrent lithogenesis and a proven non-refluxing, stone-draining CDF (as in this case), conservative management with close surveillance may be a safe and appropriate strategy, rather than mandatory invasive intervention to close the fistula (8).

### 3.4 Limitations

This study has several limitations. First, it is a single case report with a retrospective design, and the documentation of the 22-year asymptomatic interval relies on the patient's medical history and recall. Second, we cannot definitively confirm that every recurrent stone during the follow-up period was expelled through the fistula, rather than passing naturally through the ampulla of Vater. Nonetheless, the strong temporal correlation (onset of the long-term asymptomatic period immediately after the biliary surgery that possibly caused the fistula), the causal relationship between the unique anatomical configuration and the observed protective mechanism, and the absence of any alternative explanation for the 22-year remission in this high-risk patient, collectively provide some evidence to propose the protective effect of the CDF hypothesis.

## 4 Conclusion

In rare circumstances, a choledochoduodenal fistula can function as a long-term protective mechanism rather than a pathological complication, by enabling spontaneous clearance of recurrent bile duct stones and preventing biliary obstruction and ascending cholangitis. The preservation of the physiological biliary-duodenal pressure gradient and the “pressure-dependent safety valve” mechanism formed by the slender distal CBD are the core explanations for this special benign clinical course. This observation suggests that conservative management with close surveillance may be appropriate for highly selected patients with non-refluxing, functionally beneficial CDFs.

## Data Availability

The original contributions presented in the study are included in the article/supplementary material, further inquiries can be directed to the corresponding authors.
